# Magnetic sensors using amorphous metal materials: detection of premature ventricular magnetic waves

**DOI:** 10.1002/phy2.30

**Published:** 2013-07-21

**Authors:** Tsuyoshi Uchiyama, Shinsuke Nakayama

**Affiliations:** 1 Department of Electronics, Nagoya University of Graduate School of EngineeringNagoya, 464-8603, Japan; 2 Department of Cell Physiology, Nagoya University Graduate School of MedicineNagoya, 466-8550, Japan

**Keywords:** Biomagnetic field, magneto-impedance/inductive effect, magnetocardiogram, room temperature-operated, shieldless, electric current propagation

## Abstract

The detection of magnetic activity enables noncontact and noninvasive evaluation of electrical activity in humans. We review the detection of biomagnetic fields using amorphous metal wire-based magnetic sensors with the sensitivity of a pico-Tesla (pT) level. We measured magnetic fields close to the thoracic wall in a healthy subject sitting on a chair. The magnetic sensor head was mounted perpendicularly against the thoracic wall. Simultaneous measurements with ECG showed that changes in the magnetic field were synchronized with the cardiac electric activity, and that the magnetic wave pattern changed reflecting electrical activity of the atrium and ventricle, despite a large variation. Furthermore, magnetic waves reflecting ventricular arrhythmia were recorded in the same healthy subject. These results suggest that this magnetic sensor technology is applicable to human physiology and pathophysiology research. We also discuss future applications of amorphous wire-based magnetic sensors as well as possible improvements.

## Introduction

The detection of a magnetic field enables noncontact, noninvasive measurements of electrically active biological systems. Thus far, magnetic sensors utilizing a superconducting quantum interference device (SQUID) are practically available to measure biomagnetic fields (Koch 2004; Kaneoke 2006; Stufflebeam et al. 2009; Hari et al. 2010). However, this technology requires special equipment, such as containers of cooling liquid medium and a magnetic shield. Therefore, the total sensor system is too large and expensive for personal and single laboratory use.

In order to develop a more convenient device, we employed a pulse-driven magnetic sensor with an amorphous wire. This sensor is constituted of only ordinary electromagnetic parts, and thus it can be compacted and operated at room temperature. We have improved the sensitivity of this magnetic sensor toward a pico-Tesla (pT) level, and have been exploring biological and medical applications (Uchiyama et al. 2009, 2011; Nakayama et al. 2011a,b). In this review, we explain the amorphous wire-based magnetic sensor technology along with some new recordings of cardiac magnetic waves accompanied by ventricular premature contractions. We anticipate that this technology will be useful in medicine and physiological research fields.

### Magneto-impedance/inductive effect

In 1991, a large change in inductance in response to an external magnetic field was found during the application of an AC current to an FeCoSiB amorphous metal wire (Mohri et al. 1992). This phenomenon was thus referred to as magneto-impedance or magneto-inductive (MI) effect. It is now considered that due to the skin effect, AC current of high frequency, that is, higher than 50 MHz, flows mainly through the surface of an amorphous wire, whereas only small opposite magnetic fields occur along the longitudinal axis (Mohri et al. 1995; Sanacci et al. 2004).

Another advantage of this sensor is its tremendously linear voltage response over a wide range of magnetic fields greater than the geomagnetism. Thus, unlike SQUID sensors, amorphous wire-based magnetic sensors can be used without any magnetic shield. Also, the intrinsic magnetic field noise is estimated to be less than 10 fT/√ (Hz) even in a 10 mm wire of 30–40 μm diameter at room temperature, assuming the electron spin density of a typical Co-rich amorphous metal (Melo et al. 2008). Therefore, initially, it was expected that a highly sensitive magnetic sensor of micrometer size could be developed (Mohri and Honkura 2007). Now, 6D motion sensors and 3D electronic compasses with a sensitivity of μT level are manufactured for ‘smart’ phones.

### Magnetic sensor system

Initially, amorphous wire-based magnetic sensors were operated by applying an AC current. However, later, in order to use amorphous wire-based magnetic sensors in digital tools, a hybrid MI sensor was developed to apply excitation pulses to amorphous wires through CMOS IC. In CMOS inverters, switch-on pulses rise within several ns, being equivalent to an AC current of several tens of MHz (Mohri et al. 2002; Mohri and Honkura 2007). In line with the pulse-driven technology, we created a gradio-sensor system of amorphous wire-based magnetic sensors for biological use, by adjusting the size of the sensor element and circuit parameters.

**Figure 1:**
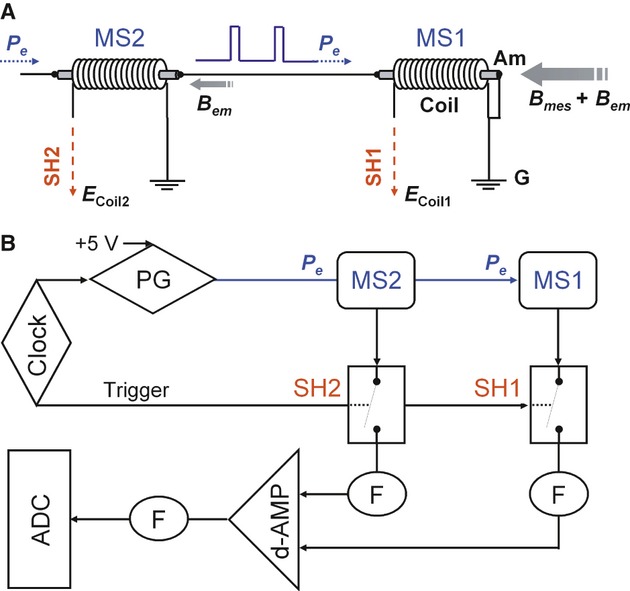
Schematic diagram of a pulse-driven magnetic sensor with an amorphous wire. (A) Magnetic sensor elements (sensor head: MS1, MS2). Each MS element is constructed with an amorphous wire (30 μm in diameter, 5 mm in length) surrounded by a transducer coil (1 mm diameter, 200–500 turns). In this gradio-sensor system, MS1 is used to measure biomagnetic activity (*B*_mes_) and MS2 is used to cancel environmental noise (*B*_em_). The two MS elements are mounted on a straight line with a distance of ∼50 mm between them. The amorphous wires of MS1 and MS2 are electrically connected, and receive the same driving electric pulses (*P*_*e*_: 5V, 10–100 nsec duration) from a pulse gate CMOS IC (PG). (B) Excitation and detection circuit diagram. A clock CMOS IC triggers PG and two sample-and-hold detectors (SH1, SH2) at ∼1–2 μsec intervals. A set of operation amplifiers (d-AMP) differentiate the voltage sampled in SH2 from that in SH1 to detect biomagnetic activity measured close to MS1. The magnification of d-AMP was ∼1000-fold. A set of high- and low-pass filters (F) was mounted along with d-AMP, acting as a band-pass filter between 0.3 and 40 Hz. The biomagnetic activity is recorded in a computer through an analog-to-digital converter (ADC).

Figure 1 shows a block diagram of an amorphous wire-based magnetic sensor system equipped with a pair of magnetic sensor elements, operating as a gradiometer in which one magnetic sensor detects cardiac magnetic activity and the other cancels environmental noise (Uchiyama et al. 2009; Nakayama et al. 2011a). A clock IC triggers a pulse gate IC (PG) that applies an excitation pulse (*P*_e_: 5V, 10–100 nsec) to amorphous wires in a pair of magnetic sensor elements (MS1, MS2) at ∼1–2 μsec intervals: A set of CMOS IC is used as a multivibrator circuit. Each magnetic sensor element is made up of a transducer coil (1 mm diameter, 200–500 turns) and a CoFeSiB amorphous metal wire (∼30 μm in diameter, 5 mm in length). The same clock IC simultaneously triggers a pair of sample-and-hold circuits (SH1, SH2) to detect the peak amplitude of the induction potential in the transducer coils of MS1 and MS2 (*E*_Coil1_ and *E*_Coil2_, respectively). A set of operation amplifiers (d-AMP) is used to differentiate the peak amplitude of *E*_Coil2_ from *E*_Coil1_, corresponding to the subtraction of MS2 from MS1 magnetic fields to cancel environmental magnetic noise, and to amplify the subtracted signal ∼1000-fold. Together with the operation amplifier system, high and low cut filters (Fs) are applied to transfer electric signals between 0.3 and 40 Hz. The resultant output voltage is recorded in a computer through an analog-to-digital converter (ADC) with 14 or 16 bits. Positive magnetic signals in figures represent magnetization toward the MS1 sensor head. This magnetic sensor system achieves a sensitivity of ∼88 μV/nT (∼0.11 V/A/m, i.e., B = μ_0_H and μ_0_ = 1.256 × 10^−6^ T/A/m) with an almost completely linear voltage conversion of the magnetic field within the range of ±9.4 μT. Also, this sensor system enables quasi-real time recordings of biological magnetic fields, because the clock CMOS IC operates GP and SH circuits at ∼1–2 μsec intervals.

## Methods

In healthy volunteers, cardiac magnetic activity was measured using the amorphous wire-based magnetic sensor system. No magnetic shield equipment was used. Procedures of magnetic measurements were approved by an institutional committee (Nakayama et al. 2011b). Participants wore a cotton shirt less than 2 mm thick, and sat on a chair during measurements.

**Figure 2:**
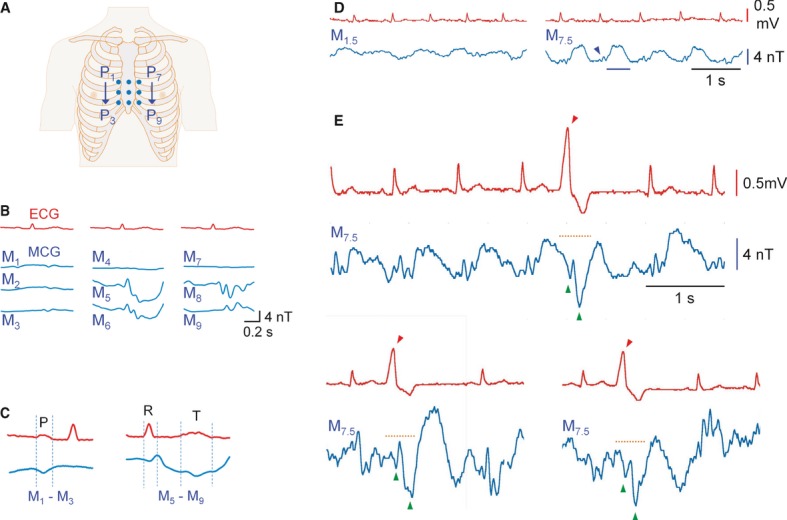
Magnetic fields measured on the surface of the human chest. (A) The positions of magnetic field measurements in B are indicated as P_1–9_. P_2_ and P_8_ are close to V_1_ and V_2_ positions (in the 4th intercostals region) in ECG, with a distance of ∼3 cm. The MS1 element was fixed on a plastic arm, with the amorphous wire perpendicularly against the thoracic wall of a subject with a distance of ∼2–3 mm. (B) Spatial distribution of magnetic activity, M_1–9_, measured in a participant measured at P_1–9_. Limb lead I ECG was simultaneously measured to average magnetic signals during three consecutive cardiac cycles. (C) M_1_–M_3_ and M_5_–M_9_ are shown. Note magnetic waves corresponding to ECG waves, that is, P wave, QRS complex, and T wave. (D) Quasi-real time measurements of magnetic waves (M_1.5_ and M_7.5_) along with ECG (limb lead I). The regions of measurements are the centers between P_1_ and P_2_ (P_1.5_) and P_7_ and P_7_ (P_7.5_) in a different participant. The arrow head and bar (blue) indicate a magnetic transient corresponding to the QRS complex and an ensuing slow magnetic wave, respectively. (E) Biphasic magnetic waves (a pair of green arrow heads) corresponding to premature ventricular contraction (PVC) (red arrowhead and dotted line). The magnetization toward the sensor head is positive. B and C are modified from Figure 3 in Nakayama et al. (2011b).

Spatial distribution of cardiac magnetic activity was assessed at nine (3 × 3) matrix points on the surface of the thoracic wall (Fig. 2A). Measurement positions 2 and 8 (P_2_ and P_8_) were near V_1_ and V_2_ in ECG (4th intercostals on the right and left lateral sternal lines), respectively, and adjacent positions were ∼3 cm apart. During measurements, the MS1 sensor head (element) was placed perpendicularly against the thoracic wall of the subject at a distance of ∼2 mm. As respiratory states largely alter the shape of magnetic waves, presumably due to changes in the position of the heart (Nakayama et al. 2011b), the subject's breath was held under shallow inspiration.

## Results

Cardiac electric activity was simultaneously monitored using limb lead I ECG, and was used to average magnetic waves. The amplitude of magnetic wave was maximal near the center (P_5_) or the middle left (P_8_) in MCG (Fig. 2B). On the other and, in ECG, when separately measured, the amplitude increased progressively toward the bottom left (P_9_). This difference suggests that the measured magnetic waves reflected cardiac magnetic activity and are not solely due to a local electric current on the surface of the thoracic wall. Moreover, arithmetic calculations between magnetic signals indicated that atrial and ventricular magnetic activity could be measured separately, unlike ECG recordings, which always contain electric activity of both the atrium and ventricle. Subtraction of the MS1 signal in P_3_ from that in P_1_ (M_1_–M_3_) (Fig. 2C, left panel), and M_5_–M_9_ (right panel) provided with magnetic waves, which reflected atrial (P wave) and ventricular (QRS complex along with T wave) electric activity, respectively.

Similar location-dependent magnetic waves were observed in another participant. The left and right panels in Figure 2D (M_1.5_ and M_7.5_) were measured in the middle of P_1_ and P_2_, and in the middle of P_7_ and P_8_, respectively. The former (M_1.5_) contained slow magnetic waves preceding the QRS complex, presumably reflecting the P wave in ECG. On the other hand, the latter (M_7.5_) consisted of a small magnetic transient corresponding to the QRS complex, followed by a large slow wave (blue arrowhead and bar, respectively). Coincidently, magnetic waves corresponding to premature ventricular contraction (PVC) in ECG (red arrow) were recorded during measurements of M_7.5_ waves in the same participants (Fig. 2E). During the period of PVC (dotted line), biphasic magnetic waves (a pair of green arrowheads) with a course slower than normal magnetic waves occurred. This could be explained by slower propagations of depolarization and repolarization through the ventricular muscle. The similar shape of three magnetic waves implied a single focus of PVC. Furthermore, the magnetic waves accompanied by PVC are rather larger than normal magnetic waves. This comparison suggests that cardiac contractility does not significantly affect amorphous wire-based MCG on the surface of the chest, because smaller pressure occurs in PVC.

## Discussion

Amorphous wire-based magnetic sensors are operated at a room temperature without using a magnetic shield, and can be made compactly. Thus, numerous possible applications are suggested in biology and medicine, especially for the analyses of integrated cellular actions, such as physiome studies. Recently, two other magnetic sensors operated at a room temperature have been reported to be available for detecting biomagnetic activity. Magnetic sensors using integrated magnetoelectric (ME) composites of, such as SiO_2_*/*Ti*/*Pt*/*AlN*/*Cr*/*FeCoSiB thin films, can be also made compactly (Marauska et al. 2012), but currently appear to be less sensitive than amorphous wire-based magnetic sensors. On the other hand, an atomic magnetometer using ^87^Rb vapor detects α-oscillations in the brain in a magnetic shield room, but the noise level increases at lower frequencies due to the instability of heater current (Sander et al. 2012). It is considered that future research and development will center these room temperature-operated magnetic sensors.

**Figure 3:**
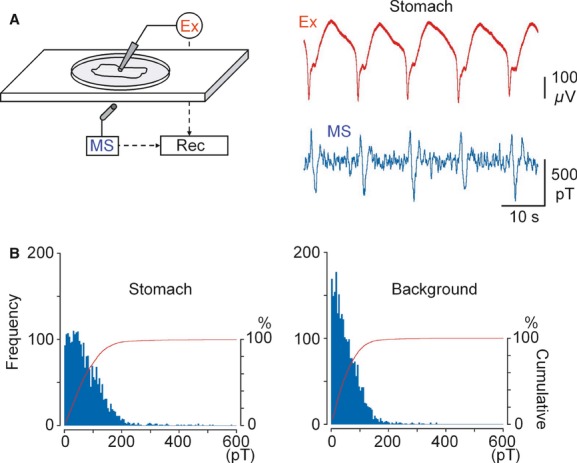
Magnetic activity measured in muscle preparations isolated from the guinea pig stomach. (A) Preparations were mounted in a recording chamber with MS1 placed below, and an extracellular electrode (Ex) was placed near the center of MS1 (left panel). An example of simultaneous measurement of spontaneous magnetic (MS = MS1–MS2: blue) and electric activities (Ex: red) (right panel). (B) Amplitude histograms constituted from magnetic recordings of the guinea pig stomach (left panel) and background noise (right panel). Bin width = 5 pT. Red curves represent cumulative (%) of the histograms. This figure was modified from Figures 3 and 4 in Nakayama et al. (2011a).

Figure 3 shows an example of measurements using an amorphous wire-based magnetic sensor in the musculatures of a guinea-pig stomach, in which special pacemaker cells are known to propagate electric activity through the tissue (Nakayama et al. 2006, 2011a). Magnetic activity and simultaneously measured electrical slow waves were well synchronized (A–B). Also, amplitude histograms distinguish magnetic activity caused by stomach pacemaker cells from background noise (C–D). Likewise, amorphous wire-based magnetic sensor technology may be used for noninvasive and aseptic evaluation of electrically excitable cellular tissues derived from stem cells. For example, gut-like small tissues derived from mouse embryonic stem (ES) cells generate giant electric potentials of ∼50 mV in amplitude. However, burst-like transients frequently occur following the repolarization phase, unlike normal gut pacemaker potentials (Ishikawa et al. 2004). Furthermore, cardiac cellular tissues derived from induced pluripotent stem (iPS) cells generate abnormal electric activities, such as long QT, when mutated ionic channels are contained (Kaichi et al. 2010; Nelson et al. 2010).

The sensitivity of amorphous wire-based magnetic sensors is currently several tens to hundreds of pT (Fig. 3C–D), and could be improved by the following procedures. First, MS elements, excitation, and detection circuits shown in Fig. 1, could be made as microelectro mechanical system (MEMS)-like integrated circuits, thereby circuit parameters may be optimized to achieve a noise level comparative to the intrinsic magnetic field noise theoretically estimated (Melo et al. 2008). Secondly, excitation and detection circuits may be replaced with digital systems utilizing rapid AD and DA converters with a frequency of several hundreds of MHz. In such systems, the whole induction potential decay in pick-up coils of MS elements is sampled. Off-line analyses enable the extraction of details of changes in biomagnetic fields. Also, digitalized systems provide a detector of static magnetic fields with high sensitivity. These sensors can be applied to medical tools, regulating the positions and angles of objects. Thirdly, amorphous metal wires, which detect external magnetic fields as a shift of internal magnetization, could be improved. Currently, Co-rich amorphous wires with a diameter of ∼30–40 μm are used. The intrinsic noise could be largely reduced in new amorphous metal materials. For stable Magnetoencephalogram (MEG) measurements, which require a sensitivity of fT, presumably amorphous wire materials need to be improved. With such improvements, amorphous wire-based magnetic sensors and other magnetic sensors operated at a room temperature, are expected to be applied in numerous fields, including human physiology and pathophysiology.
